# The Effect of Organizational Resilience and Strategic Foresight on Firm Performance: Competitive Advantage as Mediating Variable

**DOI:** 10.22037/ijpr.2021.116145.15723

**Published:** 2021

**Authors:** Mahdieh Fathi, Nazila Yousefi, Hossein Vatanpour, Farzad Peiravian

**Affiliations:** a *Department of Pharmacoeconomics and Pharma Management, School of Pharmacy, Shahid Beheshti University of Medical Sciences, Tehran, Iran. *; b *Department of Pharmacoeconomics and Pharma Management, School of Pharmacy, Shahid Beheshti University of Medical Sciences, Tehran, Iran. *; c *Department of Pharmacoeconomics and Pharma Management, School of Pharmacy, Shahid Beheshti University of Medical Sciences, Tehran, Iran.*

**Keywords:** Organizational resilience, Strategic foresight, Pharmaceutical companies, Firm performance, Competitive advantage

## Abstract

The pharmaceutical industry’s performance in the global economy has been affected by the growing competition associated with globalization, economic liberalization, and the trade-related aspect of the intellectual property rights (TRIPS) agreement. To maintain performance, organizations need to consider strategic foresight (SF) and organizational resilience (OR) to anticipate future trends and survive crises. By proposing a conceptual framework, this study examines the relationship between organizational resilience, strategic foresight, competitive advantage (CA), and firm performance (FP). A conceptual framework was developed to assess the hypotheses in the pharmaceutical industry. Then, partial least squares structural equation modeling (PLS-SEM) was applied to investigate the relationships quantitatively. The results of structural equation modeling (SEM) based on the data generated from 202 completed questionnaires by the pharmaceutical companies in Iran demonstrate that OR, SF, and CA have significant positive impacts on FP. Moreover, CA partially mediates the relationship between OR and FP and also between SF and FP. The findings of this study enrich the existing literature by demonstrating that early detection of environmental change and resilient manner assist Iranian pharmaceutical firms to survive if joining the WTO. This is the first study that examines the direct and indirect effect of OR and SF on the FP, considering the mediating impact of CA. This investigation attempts to address the mechanisms through which OR and SF affect organizational performance, especially in the pharmaceutical industry.

## Introduction

Organizations are often confronted with challenges due to the quick changes in the business environment, such as emerging technologies, variations in customer preferences (1), and shifts in the socio-cultural, political, and legislative environments (2–4).

These rapid changes in business, society, and the world have redoubled the need to study resilience in different industries in order to understand and respond to the crisis; therefore, the concept of resilience within organizations has been emphasized by researchers (5, 6). 

The available literature indicates that resilience is one of the most important inherent features of an organization to survive and thrive in a changing, complicated, and uncertain business world (7, 8). Organizational resilience (OR) assists the organization in overcoming the obstacles stemming from environmental threats and risks, enhancing the likelihood of project success (9), and allowing the organization to continue its performance during both normalcy and crises (10).

In addition to resilience, companies have started to use strategic foresight (SF) to anticipate changes to better respond to them and minimize uncertainties (11, 12). SF is defined as firms’ structural and cultural capabilities to detect changes, interpret the consequences, and generate effective responses (13). 

The competitive position of firms is influenced by high levels of environmental, technological, and demand uncertainties (14). Therefore, timely anticipation of changes and rapid adaptation lead to competitive advantage (CA) and, consequently, success in the marketplace (15). 

Our study focuses on the pharmaceutical industry since it has undergone a tremendous change recently due to advances in technology and Trade-Related Aspect of Intellectual Property Rights (TRIPS) (16). Furthermore, globalization and economic liberalization are significant challenges for pharmaceutical companies in developing countries. While competition is rapidly increasing, pharmaceutical companies need to behave speedily for sustainable competition. It is necessary to pay attention to the items affecting the competitiveness of the pharmaceutical industry in the international market due to the complex and competitive environment (17). However, concerning the ease of access to the global markets, there are opportunities for companies with dynamic capabilities and CA.

The World Trade Organization (WTO) is an organization with a considerable impact on foreign trade and, consequently, the economic structure of countries; however, some countries, including Iran, have not yet become a member of this organization. Therefore, Iran’s WTO accession is an enormous environmental change for the country’s local pharma industry. Accordingly, we focus on OR, SF, and the CA of Iran’s pharma industry in this situation.

Although OR is not a new concept in the literature, there is still a lack of integrated structure for its measurement and relations with other concepts for firm performance (FP). The literature emphasizes that it is difficult to understand an organization’s resilience before it is tested through the crisis. In addition, there is a gap in understanding the relationship between organizational resilience, competitiveness, and profitable performance through an integrated structure (18).

In light of these research lacuna and the importance of pharmaceutical products, this paper attempts to measure Iran’s pharma industry resilience and examine the effect of OR and SF on the performance of pharmaceutical companies considering the mediating role of CA.

## Experimental

*Theoretical background and hypothesis development*

At present, one of the main objectives of organizations is adapting to local and international shifts to maintain function (19). In this regard, OR has been recognized as the organization’s capability to anticipate and resist incidents by adapting to them and undergoing natural recovery (20).

Resilience is a multidisciplinary concept used in many different areas, including ecology (21), metallurgy (22), individual and organizational psychology (23, 24), supply chain management (25), strategic management (1), and safety engineering (26). Despite contextual discrepancies in using the term, the concept of resilience is closely related to an element’s ability to return to a steady state after a disruption. Considering these attributes, the definition of resilience does not change dramatically when applied to an organization (27). 

McManus *et al.* (2008; p. 82) defined organizational resilience as “a function of an organization’s overall situation awareness, management of keystone vulnerabilities, and adaptive capacity in a complex, dynamic, and interconnected environment” (28).

Unstable environments are creating frequent challenges. Occasional shocks or periodic revolutionary changes would happen in even relatively stable markets. Only flexible, agile, and dynamic organizations would thrive during disruption and continuously evolving marketplace environments (29). 

Koronis and Ponis (2018) have stated that there are three different approaches to resilience, including strategic resilience, i.e. having the capacity for change without first experiencing a crisis; functional resilience, which entails the capacity to survive and recover after experiencing a crisis; and people resilience, which refers to the individual and group behavior in response to the crisis (30).

The strategic resilience approach is suitable for measuring and evaluating an organization’s resilience before a crisis happens. According to this approach, timely anticipating changes and appropriate actions before the crisis protects the organization from potential risks. 

In addition, foresight can help identify emerging concepts, trends, ideas, and weak signals (13) to minimize uncertainties or risks (31).

*Organizational resilience, competitive advantage, and firm performance*

The ability of an organization to create a defensible and distinct position compared to its rivals is called CA (32–34). The resources which provide a CA for a firm should be valuable, scarce, unique, and irreplaceable (35, 36).

Two types of competitive strategies suggested by Porter (1980) are differentiation strategy and cost‐leadership strategy (37). Empirical studies argue that combining differentiation and cost-competitive strategies significantly contributes to FP. In their empirical study, Khan *et al.* (2019) demonstrated a positive association between sustainable CA and FP (38). The link between CA and FP is supported by some other studies, *e.g.* Majeed (2011), displaying that a more advanced CA leads to a higher level of performance (39). A similar finding was reported by Rahim and Zainuddin (2019) concerning the Malaysian automotive industry (40).

The competitive position of firms is substantially influenced by environmental, technological, and demand uncertainties; therefore, various levels of CA result from different levels of the risk management capacity of these uncertainties. Proactive risk management via more consideration of risk and its implementation to avoid unexpected events leads to CA (41). The firms with superior flexibility are more likely to gain and maintain their CA (14). The study conducted by Sharma et al. (2020) signifies that developing individual resilience between employees, teams, and the systems and organization’s processes to increase organizational effectiveness can create a CA in intelligent organizations. They believe that in today’s competitive circumstances, organizations with resilient employees, systems, and processes can better adapt to the varying demands of their market and increase CA, directly improving the performance of an organization (42).

The management literature has emphasized the influence of resilience on firms’ performances (1, 6, 43). Comfort *et al.* (2001) stated that organizational performance repeatedly declines in an environment with increasing complexity. To develop a risk reduction strategy in uncertain environments, they suggest that a system strike a balance between anticipation and resilience (44). While previous studies have indicated that post-crisis recovery strategies affect the performance of an organization (45), the impact of OR on business performance before the crisis is an underexplored area, especially among pharmaceutical companies.

Hence, the following hypotheses are proposed:

H1: OR positively associates with the CA.

H2: CA positively associates with FP.

H3: OR positively associates with FP.

H4: CA mediates the relationship between OR and FP.

*Strategic foresight, competitive advantage, and firm performance*

Different businesses within the global economies are affected by the severe competition following globalization and economic liberalization (46, 47). 

The foresight program empowers organizations to identify and respond to emerging opportunities in markets and technologies and, consequently, create a sustainable CA (48). 

Napitupulu (2018) believes that firms’ strategic foresight can achieve sustainable CA in globalization and boundary-less trade (49). SF would lead to an overall increase in a firm’s competitiveness by early detection of external variation (50). 

Many studies have empirically demonstrated that SF affects FP (12, 51–53). SF contributes to a better performance in an organization through understanding the emerging risks and business opportunities, drivers, incentives, and causalities related to future opportunities and alternative decisions (54).

Given the above explanations, we have proposed the following hypotheses:

H5: SF positively associates with CA. 

H6: SF positively associates with FP. 

H7: CA mediates the relationship between SF and FP.

**Figure 1:**
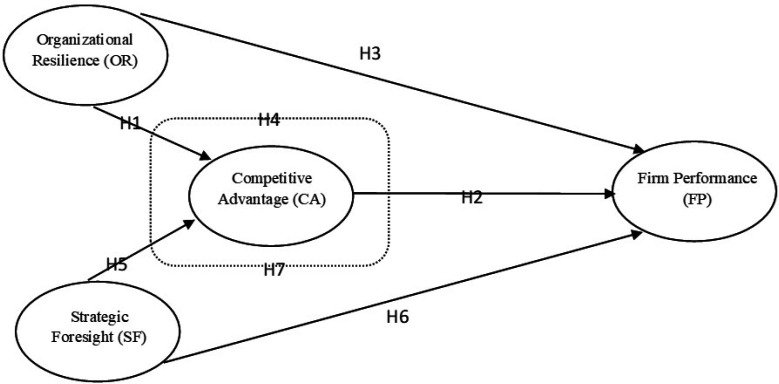
Conceptual model

The proposed conceptual model is shown in Figure 1. 

## Method

In this part, we first describe the research model and the measures used in this study. Then, we discuss the research instrument and its validation. Finally, sampling, data collection, and data analysis procedures will be explicated. 

*Study approach*

This research has employed an empirical cross-sectional survey using a validated questionnaire. The research object was Iranian export-oriented pharmaceutical manufacturers. Pharmaceutical industry faces high uncertainty and competitiveness, especially in a liberalized and globalized economy. OR and SF are highly important for such firms to maintain competitiveness and improve their performance. According to the Iranian Human Pharmaceutical Industry Owners Syndicate website, the sample comprised all Iranian export-oriented pharmaceutical manufacturers in 2019. 

The respondents were senior managers and department managers of Iranian export-oriented pharmaceutical companies. Five hundred questionnaires were distributed among 47 firms via e-mail, and by a 40% effective response rate, 223 filled questionnaires were received. Out of this, 21 surveys were discarded since they were incomplete. 

As mentioned, this research employs Structural Equation Modelling (SEM) for statistical analysis. SEM is widely used to quantify and test substantive theories in many scientific disciplines such as sociology, biology, and economics (55). In an attempt to evaluate the model in this study, PLS was selected as a component-based approach of SEM, which, as claimed by Hsu *et al.* (2006), is a convenient tool for analyzing quantitative data, particularly for small sample sizes (56). 

*Measurement of variables*

The questionnaire of the current study has five sections. The first part of the survey includes the descriptive data of companies (including the number of employees and founding year); as shown in Table 8, the second part contains the measurement of OR; the third part entails the measure used to assess SF; the fourth part contains the CA questionnaire, and the fifth part is the measure of FP.

The scale devised by *Lee et al.* (2013) was used to measure organizational resilience. It contains 30 items, tapping into three subscales including leadership and culture (measured by sixteen items), networks (measured by five items), and change-ready (measured by nine items) (18). To gauge the construct of strategic foresight, the scale developed by Paliokaite and Pacesa (2014) was employed. Eight items in this questionnaire measure environmental scanning, while three of them assess strategic selection (13). Some minor modifications were performed on the wordings of the questionnaire statements.

Measures for the dimensions of CA were drawn from previous studies (57–60). We consulted academicians and experts from the pharmaceutical industry to ascertain the content validity of these measures.

The FP questions were determined based on the balanced scorecard (BSC) categorized into four main perspective: financial performance, customer performance, internal business processes performance, and learning and growth performance. A 5-point Likert scale ranging from ‘strongly disagree’ to ‘strongly agree’ was used in this research.

## Results

*Sample profile*

The demographic information related to both firms and respondents’ main characteristics is shown in Table 2. About 26% of respondents had less than ten years, and about 10 percent had over 30 years of working experience in the pharmaceutical industry. A total of 71.4% of the firms had more than 250 workers, and all selected companies had over 15 years of experience in pharmaceutical production.

*Non-response bias and common method bias*

In order to check for non-response bias, the early and late responses were compared as recommended by Armstrong and Overton (1977) (61). The result of the t-test did not show a significant difference between the early and late response groups, hence the lack of non-response bias in this study. Harman’s one-factor test was carried out to assess the common method bias (62). It revealed that only 24.70% of the total variance was defined by the extracted factor, which is less than 50%. The above results indicated that non-response bias did not occur in this study.

*Validity and reliability analysis*

The reliability of the individual items of the research construct was assessed by using the factor loading method. According to the results in Table 1, all factor loadings ranged from 0.706 to 0.837 and surpassed the recommended 0.7 thresholds, confirming construct validity for all constructs in the research framework. Then, the model’s internal consistency was evaluated by calculating the composite reliability (CR) and the Cronbach’s α (63). The results indicated that composite reliability and Cronbach’s α coefficients were greater than the suggested threshold of 0.70 for all of the constructs, demonstrating adequate total internal consistency. 

In addition, convergent validity was checked to see whether the measurement scales truly measured the corresponding constructs. The concurrent validity was assessed by calculating the average variance extracted (AVE). The results showed that the score of AVE for all constructs exceeded the recommended value of 0.5 (64–66).

The Fornell-Lacker criterion was used to evaluate the discriminant validity of the research constructs. According to this method, the square root of each construct’s AVE should be larger than its correlation with other latent constructs. As illustrated in Table 3, the constructs were significantly different from each another.

*Hypothesis testing results and the mediating effect*

**Figure 2:**
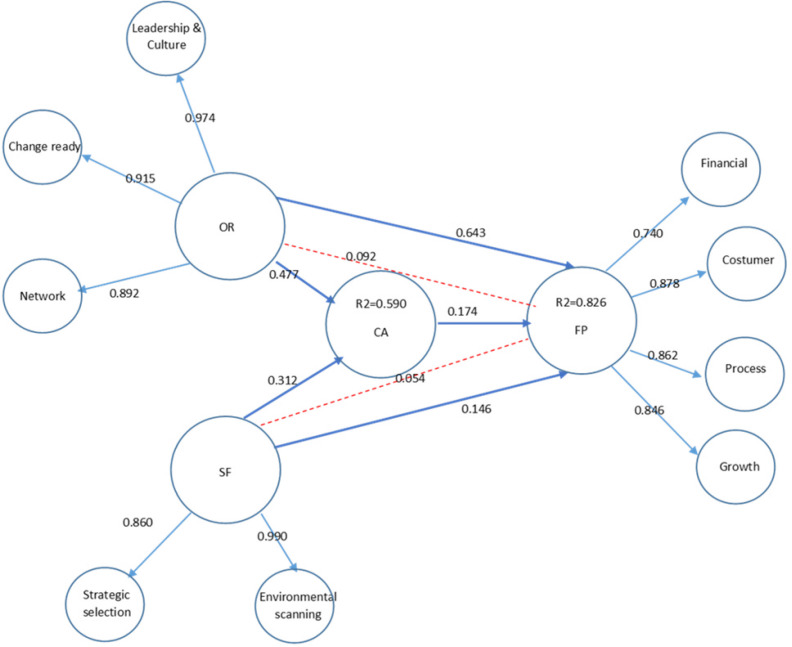
Structural model of the impact of organizational resilience and strategic foresight on performance with the mediating effect of competitive advantage

As previously mentioned, the direct and indirect effects of OR and SF on CA and FP were examined in this research. Figure 2 demonstrates the standardized path coefficients for all endogenous and exogenous constructs and the coefficient of determination (R2) for endogenous constructs. According to the results shown in Figure 2 and Table 4, the standardized coefficient for the path between OR and CA was 0.47 (*t*-value = 7.56, *p < *0.000), confirming that OR positively and significantly contributed to CA (H1). Moreover, the standardized path coefficient between CA and FP was 0.17 (*t*-value = 3.92, *p < *0.001), corroborating the positive and significant direct effect of CA on FP (H2). It was also discovered that OR had a positive and significant direct influence on FP (*t*-value= 12.61, *p < *0.000) (H3). However, the coefficient for the direct effect of OR on FP was 0.643, whereas that of the indirect effect through CA was 0.092 referred to Table 5. Hence, the results partially supported H4.

This study also revealed that SF positively and significantly affected CA (H5) with the standardized path coefficient of 0.32 (*t*-value = 5.84, *p < *0.000) and FP with the standardized path coefficient of 0.14 (*t*-value=3.37, *p < *0.019) (H6). Conversely, the path coefficient for the direct effect of SF on the FP was 0.146, whereas that of the indirect effect of SF on the FP through CA decreases to 0.054 referred to Table 5. Therefore, the results partially supported H7. 

In addition, the robustness of findings was examined on the company background characteristics such as firm age, size, and export value as control variables. Table 7 indicates that size and export value significantly influenced CA and FP. Based on the results, age exercised no significant impact on FP but had a significant negative effect on CA. 

The goodness-of-fit of the estimated model was evaluated by the coefficient of determination (R2), for the endogenous variables. As indicated in Table 7, 82.6% of the variance in FP is explained by CA, OR, and SF, which indicates a strong fitness. Besides, 59% of the variance in CA was explained by OR and SF. Obtaining the value of 0.736 indicates a strong fit of the model based on Wetzels *et al.* (2009) GOF classification (67). Furthermore, to demonstrate the path model’s predictive accuracy, the predictive relevance (Q2) was also evaluated (68). The predictive accuracy of the model is considered convenient if Q2 values are more significant than zero for the endogenous construct. According to the results, Q2 values for CA and FP were 0.314 and 0.452, respectively, demonstrating high predictive relevance in terms of the endogenous construct (68).

## Discussion

The pharmaceutical industry’s performance in the global economy has been affected by the growing competition associated with globalization, economic liberalization, and the TRIPS agreement. Organizations need to consider SF and resilience to maintain performance to anticipate future trends and survive during a crisis. This study provides an understanding of the relationship between OR, SF, CA, and FP by proposing a conceptual framework. Although previous research has separately investigated the impacts of the OR or SF on the CA, CA on FP, and the OR or SF on FP, we did not find any integrated model showing all relations simultaneously, especially in the pharmaceutical industry. Furthermore, due to the scarcity of empirical research, it is difficult to understand an organization’s resilience before a crisis occurs. 

The findings of the current study showed that OR positively affects CA (H1). These findings align with Webb’s (2006) study, which suggested that resilience can be considered a source of CA (69). It was also discovered that OR has a positive and significant effect on FP; hence, H3 is supported. This finding is in line with the study conducted by Suryaningtyas *et al.* (2019), who stated that OR significantly influences FP. (70). Therefore, it is worth discussing the mechanisms through which OR and SF affect organizational performance, especially in the pharmaceutical industry. The components of OR– leadership and culture, networks, and change-ready – can enable such companies to adapt to the changes and react properly to maintain their performance. By considering the mentioned issues, it is evident that OR and its dimensions significantly contribute to the pharmaceutical companies’ financial and non-financial performance.

Moreover, our study concludes that CA positively and significantly affects FP (H2) and partially mediates the relationship between OR and FP (H4). This is consistent with prior studies; Khan (2019) endorsed that sustainable CA has a significant positive relationship with FP (38). Based on the present study’s findings, the effect of OR on FP declines when CA mediates it. It can be argued that the lack of sustainable presence of foreign pharmaceutical companies in Iran caused by tariffs, on the one hand, and the weak GMP standards of Iranian pharmaceutical companies and consequently their inability to expand their market share in the global market, on the other hand, have led to the poor competition of Iranian pharmaceutical companies. Joining the WTO is a suitable way to improve the competitive environment for Iranian pharmaceutical companies. According to Rahimi (2011), improving technical knowledge, following international guidelines and standards, enhancing competing abilities, and finding better-exporting markets are some of the benefits reaped through joining the WTO (71).

Some previous studies highlighted the positive impact of SF on CA and FP (50, 52, 54 and 72–74). Similarly, the findings of this study indicated that SF positively and significantly affect CA and FP, lending support to H4 and H5. The findings illustrate that proactive behavior leads to organizational survival and stability in adverse situations and mitigates the organization’s negative social and economic effects.

Furthermore, CA partially mediated the relationship between SF and FP (H7). The results of the current study demonstrated that the effect of SF on FP declines if it is mediated by CA. SF aims to predict the future for quickly responding to changes and improving the FP. Still, due to the lack of a strong competitive environment, the indirect effect of SF on FP through CA is less than the direct one.

The present study also explored the effect of control variables, including age, size, and export value of pharmaceutical companies, on the CA and FP. The results revealed that FP is positively affected by the size and export value of the pharmaceutical companies but is not influenced by the company’s age. Various studies have examined FP and have attested to the significant impact of the company’s size as a control variable on FP (75, 76). On the other hand, previous research has shown that age-related organizational competencies can contribute to FP, so older firms perform better than younger firms (77). In contrast to the previous research findings, the results obtained in this study indicated that performance is not significantly affected by the company’s age.

According to the present study, age, size, and export value significantly impact the CA of pharmaceutical companies. This result follows prior studies showing that larger firms can better use internal resources to gain a higher CA (78). The results of this study indicated that age negatively affects CA. In Iran, older pharmaceutical companies have less CA than younger companies due to the older equipment, higher production cost, and weak GMP standards.

As a result, organizations involved in detecting the external changes, disruptions, and emerging trends by SF and proactively making adjustments in the face of challenging conditions employing resilience can gain a CA and improve their performance.

Our study expands the literature by demonstrating that the early detection of environmental change and resilience help Iranian pharmaceutical firms survive upon Iran’s WTO accession.

**Table 1:** Measurements of variables

**Constructs**	**Variables**	**No. of items**	**Factor loading**
	Leadership and culture: leadership Staff engagement Situation awareness Innovation Networks Change ready: Rules and regulation Unity of purpose	- 4 5 4 3 5 - 4 5	- 0.711-0.817 0.716-0.775 0.707-0.836 0.765-0.831 0.706-0.783 - 0.751-0.793 0.736-0.769
	Environmental scanning Strategic selection	8 3	0.742-0.829 0.707-0.852
		8	0.728-0.785
	Financial perspective Customer perspective Internal process perspective Learning and growth perspective	3 3 3 3	0.771-0.800 0.706-0.727 0.707-0.787 0.717-0.805

**Table2:** Sample profile

**Respondents’ profile**	**Frequency**	**Percentage**	**Companies’ profile**	**Frequency**	**Percentage**
Level of education			Firm's age		
Bachelor	52	25.5	>20 years	6	28.6
Master	130	64.5	< 20 years	15	71.4
PhD	20	10			
Industrial working experience			Firm's size		
>10 years	53	26.2	> 250	6	28.6
10-20 years	90	44.5	250-500	9	42.8
20-30 years	39	19.3	< 500	6	28.6
< 30 years	20	9.9			

**Table 3:** Validity and reliability analysis

**Fornell-Lacker criterion**					**Cronbach’s α**	**CR**	**AVE**
	SF	OR	FP	CA			
SF	0.81				0.931	0.941	0.593
OR	0.77	0.90			0.972	0.974	0.554
FP	0.78	0.74	0.76		0.926	0.937	0.554
CA	0.71	0.75	0.74	0.75	0.885	0.909	0.557

**Table 4:** Coefficient estimates and hypothesis tests

Hypotheses		Path coefficient	t-statistic	*p*-value	Decision
H1	OR → CA	0.477	7.563	0.000	Accepted
H2	CA → FP	0.174	3.920	0.001	Accepted
H3	OR → FP	0.643	12.618	0.000	Accepted
H5	SF → CA	0.312	5.848	0.000	Accepted
H6	SF → FP	0.146	3.377	0.019	Accepted

**Table 5:** Effects of OR and SF on CA and FP

	Independent variable	Dependent variable (endogenous variables)		
			CA	FP
	OR	Direct effect Indirect effect Total effect	0.477^*^ - 0.477^*^	0.643^*^ 0.092^*^ 0.735^*^
	SF	Direct effect Indirect effect Total effect	0.312^*^ - 0.312^*^	0.146^*^ 0.054^*^ 0.200^*^
	CA	Direct effect Indirect effect Total effect		0.174^*^ - 0.174^*^

The mediating impact of CA on the relationship between OR and FP (H4) and the relationship between SF and FP (H7) is shown in this table; *p < 0.05.

**Table 6:** Effect of control variables on the estimated model

	Original Sample	T Statistics	*p*-values	Result
**Age --> FP**	0.079	1.857	0.067	Nonsignificant
**Age --> CA**	-0.200	3.824	0.000	Significant
**Export --> FP**	0.031	2.757	0.001	Significant
**Export --> CA**	0.069	2.062	0.000	Significant
**Size --> FP**	0.103	2.345	0.021	Significant
**Size --> CA**	0.210	3.050	0.003	Significant

**Table 7:** Goodness-of-fit measures

	Coefficient of determination (R^2^)	Predictive Relevance (Q^2^)	Goodness Of Fit (GOF)
**CA**	0.597	0.314	0.736
**FP**	0.824	0.452	

**Table 8:** Questionnaire items

**Organizational resilience**	
**Leadership and culture**	
Leadership	
L1	There would be good leadership within our organization if Iran joins the WTO.
L2	Our organization regularly re-evaluates what we are trying to achieve.
L3	In our organization, the staff accept that management may need to make some decisions with little consultation in a crisis.
L4	Our management thinks and acts strategically to ensure that we are always ahead of the curve.
Staff engagement	
S1	The staff know what they need to do to respond to unexpected problems.
S2	Our organization's culture is to be very supportive of staff.
S3	People in our organization feel responsible for the organization's effectiveness.
S4	Our organization has high staff morale.
S5	People in our organization are committed to working on a problem until it is resolved.
Situation awareness	
SA1	The staff interact regularly to know what's going on in our organization.
SA2	Our managers actively listen for problems.
SA3	We are mindful of how the success of one area of our organization depends on the success of another.
SA4	We learn lessons from the past and make sure those lessons are carried through to the future.
Innovation and creativity	
I1	The staff are actively encouraged to challenge and develop themselves through their work.
I2	We are known for our ability to use knowledge in novel ways.
I3	The staff are rewarded for "thinking outside of the box."
**Networks**	
EP1	We made agreements with foreign companies in order to transfer technological know-how.
EP2	We made relationships, production or distribution of products.
EP3	We can collaborate with others in our industry in the field of joint activities, including supply, production, or distribution of products.
EP4	We understand how we are connected to physicians and actively manage those links.
EP5	We understand how food and drug administration actions would affect our ability to respond, and we actively manage those links.
**Change ready**	
Rules and regulations	
R1	Current criteria for drug registration in Iran are appropriate to support domestic production.
R2	Current laws for intellectual property rights in Iran are appropriate to protect domestic products.
R3	The current pricing laws for drugs in Iran are suitable for supporting domestic products.
R4	There is enough knowledge to use the exceptions of the patent in the country, such as compulsory licensing.
Unity of Purpose	
PS1	International regulations in the field of quality assurance are implemented and enforced in our company.
PS2	Our priorities for selecting suppliers are based on providing better access to the raw materials.
PS3	We are mindful of how joining the WTO would impact our organization.
PS4	We have clearly defined priorities for what is important during and after joining the WTO.
PS5	We understand the minimum level of resources our organization needs to operate.
**Strategic foresight**	
Environmental scanning	
ES1	We have an active network of contacts with the scientific and research community.
ES2	We collect information on patents.
ES3	We are scanning in areas such as technological, political, and socio-cultural environment.
ES4	We are scanning our customers.
ES5	We are scanning our competitors.
ES6	We also scan for developments in the markets and/or industries in which we are not currently involved.
ES7	We also consider new issues, trends, and technologies whose relevance to our business cannot yet be assessed.
ES8	We plan for the medium and long term.
Strategic selection	
SS1	We use scenarios to describe potential futures.
SS2	We apply visioning methods, for example, balanced scorecard, appreciation inquiry, road-mapping.
SS3	Our company develops activity plans that optimize progress toward the organizational strategy.
**Competitive advantage**	
CA1	Our company has the competitive advantage of low cost compared to the competitors.
CA2	Our company has better proficiencies of internal market research than foreign competitors.
CA3	Our company's profitability is better than the competitors.
CA4	Our company occupies an important position in comparison with the competitors.
CA5	Our company provides higher quality products than the competitors.
CA6	We develop or use newer technologies in our products compared to foreign competitors.
CA7	Our brands have excellent customer recognition.
CA8	Our products are unique, and nobody but our company can offer them.
**Organizational performance**	
Financial	
F1	The market share of our company over the past three years is above the average of the pharma industry.
F2	The share growth of our company over the past three years is above the average of the pharma industry.
F3	The profitability of our company over the past three years is above the average of the pharma industry.
Customer	
CU1	The clients are satisfied with the company's products.
CU2	The company is responsive to customers' complaints.
CU3	The company regularly invests in customers' needs and demands.
Process	
Pr1	The internal processes of the company are adjusted to respond to customers' needs.
Pr2	The company's processes have been simplified in order to be agile.
Pr3	Future threats such as joining the WTO are considered in reforming the company's internal processes.
Growth	
Gr1	The employees are promoting in their job environment.
Gr2	The company has suitable performance in employees' education.
Gr3	The employees are satisfied with the company's environment.

## Conclusion

Today, the pharmaceutical industry is facing several challenges due to uncertainties. These challenges cause risks and vulnerabilities in the pharmaceutical industry, jeopardizing society’s health. In this paper, we shed light on the role of OR and SF helping organizations gain a CA and improve performance. A conceptual framework was adopted in this study based on existing literature, which comprised OR and SF as the main research constructs. This model was statistically validated using PLS-SEM. Relying on the previous studies, it is concluded that OR and SF directly and indirectly affect FP.

Furthermore, it is suggested that CA can lead to an improved FP and also mediates the relationship between OR and FP and the relationship between SF and FP. The findings of this study are valuable to the pharmaceutical industry stakeholders to improve resilience and mitigate local and global vulnerabilities. Pharmaceutical companies should focus on firm resilience before facing a crisis. This is because resilient organizations better understand organizational continuity and are more likely to survive during adverse events (79). The managers of pharmaceutical companies should be aware of the strengths, weaknesses, opportunities, and threats of their organization in the dynamic environment. In this regard, OR and SF can assist pharmaceutical companies in achieving this awareness and preparing for environmental change.

## Funding

This research did not receive any specific grant from funding agencies in the public, commercial, or not-for-profit sectors.

## Competing interests

The authors declare that they have no conflict of interest regarding the publication of this manuscript.

## Author’s contributions

MF designed and performed the experiments, derived the models, and analyzed the data. NY, HV and FP verified the analytical methods. NY supervised the findings of this work and investigated the data accuracy. All authors contributed to the final manuscript. 
